# “My Little Son, My Everything”: Comparative Caregiving and Emotional Bonds in Dog and Child Parenting

**DOI:** 10.3390/ani15233358

**Published:** 2025-11-21

**Authors:** Kata Mária Udvarhelyi-Tóth, Ivett Szalma, Lóránt Pélyi, Orsolya Udvari, Erika Kispeter, Eniko Kubinyi

**Affiliations:** 1 Department of Ethology, ELTE Eötvös Loránd University, 1117 Budapest, Hungary; 2 MTA-ELTE Lendület “Momentum” Companion Animal Research Group, 1117 Budapest, Hungary; 3 ELTE Centre for Social Sciences Lendület “Momentum” Reproductive Sociology Research Group, 1097 Budapest, Hungary; szalma.ivett@tk.hu (I.S.); pelyi.lorant@tk.hu (L.P.); orsolya.udvari@stud.uni-corvinus.hu (O.U.); 4 Institute of Social and Political Sciences, Corvinus University of Budapest, 1093 Budapest, Hungary; 5 HCSO Institute for Quantitative Population and Economic Research, 1024 Budapest, Hungary; 6 Department of Health Services Research and Policy, London School of Hygiene and Tropical Medicine, London WC1H 9SH, UK; erika.kispeter@lshtm.ac.uk; 7 ELTE NAP Canine Brain Group, 1117 Budapest, Hungary

**Keywords:** dog ownership, pet parenting, motherhood, childless women, human–animal bond, caregiving roles, parenting styles, social life and family roles

## Abstract

Dogs are often viewed as family members, and many owners describe them as “fur babies.” However, little is known about how women with and without children perceive and practice caregiving toward their dogs, and how these experiences relate to parenting. This qualitative study explored the meanings of dog and child caregiving among 28 dog-owning women (13 mothers and 15 childless) through semi-structured interviews. All participants had lived with their dog for at least one year. Thematic analysis identified five main themes: (1) emotional meanings and motivations of caregiving, (2) practical caregiving and daily routines, (3) responsibility and dependency, (4) social relationships and support, and (5) life course perspectives. Both mothers and non-mothers described their dogs as sources of joy, companionship, and unconditional love. Women without children often saw their dogs as child substitutes, while mothers stressed the greater responsibility and permanence of raising children. Dog ownership and parenting influenced social life and work differently: dogs often increased social interaction and offered flexibility, whereas children introduced stricter routines and reduced spontaneity. Overall, dogs fulfilled important emotional and caregiving needs, particularly among women without children, but did not replace the unique social and moral responsibilities of parenting.

## Simple Summary

Dogs are increasingly regarded as family members, and many owners describe them as their “fur babies.” However, little is known about how women who are mothers and those without children think about and care for their dogs, and how these experiences compare to parenting. To explore this, we interviewed 28 women who had lived with a dog for at least one year. Both mothers and non-mothers described their dogs as sources of joy, comfort, and companionship, often using parental language such as “mom” or “child.” Women without children more often viewed their dogs as fulfilling caregiving needs or providing emotional continuity, while mothers emphasized the greater, lifelong responsibility involved in raising children. Caring for dogs and children also shaped daily routines differently: dogs offered flexibility and social connection, whereas children brought stricter schedules and less free time. Despite these differences, both roles were described as deeply meaningful and emotionally rewarding. Overall, our findings show that dogs can meet important emotional and caregiving needs, particularly for women without children, yet they do not replace the unique experiences and responsibilities of raising children. These insights shed light on changing forms of caregiving and the growing phenomenon of “pet parenting” in contemporary society.

## 1. Introduction

Dogs were the first animals to be domesticated [1] and have long played diverse roles in human societies, from working partners to companions, as archeological findings demonstrate [2,3]. In recent decades, the significance of pets has shifted markedly, particularly in Western societies and other societies with low fertility rates [4,5,6]. The majority of owners now regard their pets as family members, often attributing them a human-like status within the family structure [6,7,8,9,10,11,12,13]. This shift has contributed to the rise in pet parenting*,* a phenomenon in which owners engage in parenting-like activities, such as establishing routines, expressing concern for the pet’s well-being, and using baby-like speech [11,14,15,16,17,18,19,20].

These broader trends are also evident in Hungary. A nationally representative survey found that 16% of owners perceive their dog as a child [21]. This occurs in a demographic context marked by declining fertility rates and increasing childlessness. The number of live births per 1000 women aged 14–49 fell from 58 in 1980 to 37 in 2024 [22], with the proportion of childless women aged over 41 having increased from 8% in 2000 to 16% in 2016 [23]. Although childlessness is becoming more common, it is often involuntary or reflects unrealized fertility intentions [24,25]. At the same time, strong pronatalist social norms continue to promote the expectation that everyone should become a parent [26].

International research has linked similar demographic and social changes to shifts in family norms, delayed parenthood, and a growing interest in companion animals as alternative or supplementary caregiving targets [6,17,19,20,27,28,29,30,31]. In this light, Hungary represents an especially compelling case: companion dogs may fulfill emotional and caregiving needs not only among voluntarily childfree adults but also among those who are temporarily or involuntarily childless, navigating a social environment where parenthood remains strongly valued.

Research further indicates that the emotional salience of pets varies across family life stages and individuals without children often invest more time and emotional energy in dog care, forming stronger emotional bonds [16,29,32,33,34,35]. Given that many people now conceptualize their dogs as children, it is essential to examine how parenting styles, attitudes, and caregiving practices toward dogs compare to those toward children, and how these differ between parents and non-parents.

Parenting, broadly defined as the set of behaviors and strategies used to nurture, guide, and socialize a dependent individual [36] has traditionally been categorized into three styles: authoritative, authoritarian, and permissive [37,38]. Comparable patterns have been identified in dog ownership. Herwijnen et al. [39] described three canine parenting styles: *authoritarian* (high demandingness, low responsiveness), *authoritative–intrinsic value-oriented* (high responsiveness and emotional attunement), and *authoritative–training-oriented* (high demandingness and responsiveness). Similar classifications were found by Cimarelli et al. [40], who identified distinct dog–owner interaction styles that correspond to human parenting dimensions and reflect owners’ personality and attitudes toward pets. Parenting style influences the attachment and behavior of dogs [16,41], and evidence suggests that childless women who identify as “dog moms” tend to adopt a balanced, authoritative style, combining warmth with structure and moderate control [42].

Dog caregiving can resemble child-rearing in practice and as an emotional experience. Previous studies show that owners often reflect on their dogs’ needs, routines, and well-being in ways similar to parents [17,43,44,45,46,47,48,49], and they frequently communicate with their dogs using interaction styles that mirror parent–child exchanges. Physiological evidence also indicates that dog–owner interaction can activate caregiving-related emotional processes in humans [50,51,52,53,54,55]. Together, these findings suggest that the dog–human relationship may engage caregiving motivations that are comparable to those involved in parenting, providing a conceptual basis for examining how women interpret and navigate dog and child caregiving in their everyday lives.

Like young children, dogs are entirely dependent on their caregivers for food, safety, and social opportunities [17]. Many owners make personal, financial, and lifestyle sacrifices to ensure their dogs’ well-being [56,57,58,59,60,61], reflecting a form of intensive caregiving or “intensive motherhood” extended to pets [62].

Turcsán et al. [48] found that the dog–owner relationship mirrors the parent–child bond in emotional closeness, nurturance, and companionship, while maintaining low levels of conflict and an asymmetric dependency dynamic. Although previous research has identified many similarities between caring for dogs and children, little is known about how life stage and caregiving responsibilities shape these attitudes and behaviors. Unlike most prior studies using quantitative surveys or behavioral tests, the present research employs a qualitative approach, exploring the perspectives of women, both mothers and non-parents, through in-depth interviews. Our aim is to examine in detail women’s perceptions and experiences of dog and child parenting to understand how caregiving experiences converge and diverge across these life contexts. We hypothesize that while participants will perceive raising a child as a greater responsibility and constraint than caring for a dog, they will also recognize notable similarities in emotional engagement and everyday caregiving practices.

## 2. Materials and Methods

### 2.1. Ethical Approval

The study was conducted in accordance with the Declaration of Helsinki, and it was approved by the Institutional Review Board of ELTE (formerly HUN-REN) Centre for Social Sciences (protocol code No. LP2021-10/2021, approved on 16 December 2022).

### 2.2. Subjects

The study involved 28 women of reproductive age who had owned a dog for at least one year at the time of the interview. To align with our focus on the perception of dogs as companions, participants were required to primarily keep their dogs indoors. Fifteen women were childless, and 13 had one or more children. All mothers except two (Veronica and Emily) had dogs before childbirth, but Veronica had dogs in her childhood.

Each participant completed a brief demographic questionnaire before the interviews (age, education, partnership status, employment, number and age of children, number of dogs).

Participants ranged from 26 to 47 years old (M = 35.4, SD = 6.1). Most resided in Budapest (n = 21). Education and labor market status largely reflected an urban, highly educated population. To ensure anonymity, all participants selected pseudonyms, which are used throughout the manuscript and in Table 1.

### 2.3. Procedure

Participants were recruited using a two-stage process: initial contacts were identified through our social networks, and subsequent participants were recruited through referrals from acquaintances using snowball sampling. Data collection proceeded iteratively and ceased when information power indicated adequacy for the study purpose and theme development.

Semi-structured interviews were conducted in person at participants’ homes or in public locations, and online when necessary, in Hungarian. Before the interviews, participants were informed about the study procedures, confidentiality, and the voluntary nature of participation, and provided written informed consent. To ensure anonymity, participants chose pseudonyms, which were used throughout the study (Table 1).

The interviews covered the following topics:

- childhood and family background,
- education and employment history,
- relationship dynamics,
- experiences and meanings of dog ownership, and
- perspectives on having or not having children.

Interviews lasted 40–60 min, were audio-recorded, and then transcribed verbatim using transcription software (Alrite, https://alrite.io/ai/, accessed on 12 October 2022). Translations from Hungarian were verified by a back-translation check.

The interviews were also used in a separate, ongoing analysis (Szalma et al., in preparation), which examined the sociopolitical and economic dimensions of reproductive decision-making among dog-owning women. The present paper focuses on the micro-level caregiving experiences, comparing dog and child parenting behaviors, emotional bonds, and perceived responsibilities.

### 2.4. Data Analysis

Interview transcripts were analyzed using reflexive thematic analysis [63], which was chosen for its flexibility and capacity to identify patterns across qualitative data. The analysis began with a process of familiarization, during which transcripts were read repeatedly and discussed to identify preliminary ideas. Next, inductive codes were generated to capture key meanings emerging from the data.

While thematic analysis is often used to develop high-level interpretive themes, the present study initially focused on the code level to remain close to participants’ original wording and to reflect the breadth of caregiving-related experiences. Each interview was independently coded by two researchers to ensure reliability, and discrepancies were resolved through discussion.

In the final phase, conceptually related codes were clustered into five overarching themes that reflected the main dimensions of the data:

1. emotional meanings and motivations of caregiving;
2. practical caregiving and daily routines;
3. responsibility, dependency, and decision-making;
4. social relationships and support; and
5. life course perspectives.

### 2.5. Coding Framework

During coding, both spontaneous statements and responses to guiding questions were analyzed. Relevant verbatim excerpts were highlighted and accompanied by short coder summaries to capture the meaning in context. Each sentence could belong to multiple codes, as several topics often overlapped within the same narrative.

Examples:

“*I have to plan all my activities outside the home so that they last no more than 8 h, to fit between the two dog walks.*” → Practical caregiving and daily routines

“*A substitute for a child and a sibling at the same time, in my opinion. Currently, my partner and I actually consider the dog almost like a child.*” → Emotional meanings and motivations of caregiving

The complete coding framework, organized into five main themes, is presented in Table 2.

## 3. Results

Thematic analysis identified five major themes demonstrating how women experience caregiving for dogs and children. While the themes are analytically different, they are closely interlinked. The results highlight both similarities and distinctions in emotional bonds, routines, and social meanings, illustrating how dog ownership can resemble or complement parenting without fully replacing it. Below, we present each theme with data excerpts that compare dog and child caregiving experiences.

### 3.1. Emotional Meanings and Motivations of Caregiving

Participants frequently described caregiving for dogs and children as emotionally rewarding. Many spoke of mutual and unconditional love as a central source of happiness. When Hailey, a mother of one child, was asked what she enjoys about keeping a dog, she answered, “*Everything—when she cuddles up to you, when she looks at you, when she’s happy to see you when you come home. I think she’s like a mirror: a reflection of how you are, of what kind of person you are. And I believe it’s worth every bit of energy to take care of them, because you basically have a furry child.*”.

Participants also mentioned subtle sensory cues that deepened their emotional connection with their dogs. For some, eye contact, described using expressions such as “*big, cute,*” or “*doe-like*” eyes, communicated affection and closeness. Others highlighted olfactory cues, such as the familiar scent of their dog, which they compared to the comforting smell of a child. These small sensory details complemented feelings of attachment but were typically mentioned as personal nuances rather than central aspects of caregiving.

The joy of witnessing development was also frequently mentioned: Iris, a childless participant, talked about her dog: “*It’s about getting to know this little creature and watching its development more closely as it learns and absorbs experiences.*” Veronica, a mother of two children, used very similar language talking about her child: “*You can see them developing—oh my God, they were completely different just six months ago.*” Both comments point to a shared sense of wonder and gratification in observing change and growth, whether in a child or a dog.

Women often described their dogs in parental terms. Maya, who did not have children, said: “*For both of us, it feels as if he were our child—we love him that much. So yes, I feel he’s my best friend, my little son, my everything, truly*.” Bianca, another childless participant, explained that, for them, the dog functioned as a “zero child,” serving as a kind of preparation for having children. She added, “*We can’t neglect her because, well, what if she were an actual child? It would impact her little life.*” Similarly, Luna, a childless participant, shared: “*It’s not that I don’t have a child—M.* [the dog] *is my child. And I really act that way with her; I worry about her, I love her, so I don’t feel any sense of something missing.*” These reflections show how dog caregiving can evoke parental feelings and roles, sometimes serving as a symbolic or emotional rehearsal for motherhood.

However, even parents who regarded their dog as a child acknowledged a hierarchy between their emotional commitments to the dog and to their human child. Nora, a mother, explained: “*Even now, I think of him as my child. But B. [her husband] recently asked me a heart-wrenching and awful question: if there was a fire and I had to choose… But a bit more realistic was when he asked what would happen if J. [their son] turned out to be allergic to dog hair. Of course, we’d try every possible trick, but if, in the end, nothing worked, then… unfortunately, well, then J. would have to win.*” Brooke, a mother of two, expressed a similar thought: “*The children come first, then the dog. But the dog fills an irreplaceable void; without them, the home just wouldn’t feel like home.*” Such accounts illustrate how caring for dogs and children can coexist but remain emotionally differentiated.

Many childless respondents used explicit parental language to describe themselves or childlike terms to refer to their dogs. Pamela noted, “*We do have Mom and Dad in our household.*” Barbara said: “*Before I leave, I usually try to soften it a bit… to make it easier for her, to reassure her that ‘Mommy is leaving now.’*” Ella also referred to a “godmother” and a “grandmother” for her dog. Lisa said, “*My little dog is very fussy… she is a real mama’s girl.*” The linguistic parallels suggest that pet ownership provides a socially acceptable and emotionally rewarding context in which individuals can express nurturing behaviors and caregiving identities.

While many childless participants described their dogs in parental terms, others explicitly distanced themselves from equating dog care with motherhood. Margaret noted that although her dogs lived indoors and were deeply loved, “*they’re not my little children*.” Iris similarly rejected parental framing, explaining that she viewed dog ownership as an active hobby involving training and agility work rather than a substitute for parenting. Participants who engaged in structured activities such as sports or obedience training often described a stronger sense of partnership with their dogs, although this pattern was not consistent across the sample.

Willow, who wanted children but remained involuntarily childless, offered a more ambivalent perspective. She resisted the idea of her dog as a direct “*child substitute,*” yet acknowledged that caring for him helped her cope with involuntary childlessness: “*having a dog really helped me accept that I didn’t have children*”. Together, these accounts show that women navigate dog–child comparisons in nuanced ways, balancing emotional closeness with an awareness of species and role differences.

Several women also described their dogs as sources of emotional healing. Brooke, a mother of two, for instance, reflected that the unconditional love her dogs provided helped fill emotional gaps, “*a lack of love*”, experienced as a child. She also added: “*They trust me so completely and are so incredibly attached to me, and that’s such a miracle.*” Such experiences suggest that dog caregiving can serve not only as a meaningful relational role but also as a form of psychological support and self-repair.

Altogether, these reflections illustrate the emotional depth and complexity of women’s relationships with their dogs. For many, caregiving evokes feelings of love, responsibility, and purpose similar to those associated with parenting, while also revealing ambivalence and boundaries that distinguish human from non-human care.

### 3.2. Practical Caregiving and Daily Routines

Joint activities, such as walking with dogs or playing with children, were also described as emotionally rewarding. Brooke, mother of two, said, “*When they’re happy, it makes me happy: playing, doing board games together, walking in the woods.*” Similarly, Barbara, who did not have children, shared: “*It’s about paying attention, making sure he is okay, giving him treats, spending time with him, playing, teaching him…; Having to go on walks with my dog really helps me stay mentally balanced.*” Expressing the shared joy suggested that both child and dog care are experienced as emotionally reciprocal activities that contribute to the caregiver’s well-being.

Caring for both dogs and children shaped daily life through structured routines. Participants described feeding, walking, and playtime schedules similar to child-centered daily organization. As Lisa, a childless owner, explained: “*In the morning, we take her out, then work, then another walk, and if there’s no work, we plan some activity with or without the dog.*” Parents, such as Rebecca, reported integrating dogs and children into shared routines: “*We wake up, let the dogs out together with the boys; they already know this routine, an hour of play in the garden.*” Ella, who did not have children, noted that their dog’s bedtime routine mirrors that of a small child: “*We always wish her good night, and I have my own little bedtime rhyme for her… I say it to her every evening and give her little kisses on the head.*” Routines, whether morning walks or bedtime rituals, seem to foster a sense of family coherence and predictability.

Several respondents noted giving their dogs gifts on special occasions. Lily, a mother, said: “*If I have time, I make things like a dog cake or dog biscuits. She always gets a present at Christmas, too.*” Maya, who was childless, added: “*We always give him presents for Christmas and for his birthday, and she gets a little cake, too. At Christmas, she also has this ugly Christmas sweater that we put on her.*” These symbolic gestures demonstrate how dogs are integrated into family traditions, further blurring the boundary between pet care and parenthood.

While participants emphasized the joys of caregiving, they also discussed the challenges associated with both dog ownership and parenthood. Many spoke about the time commitment and the constant need for attention. Ella, a childless participant, explained that adjusting her daily schedule around her dog could be exhausting: “*It just structures your day, that you have to go home… And if there’s an evening event in the city, who’s going to do the 50-min journey there and back just to let her out for 15 min?*” However, caring for a child was described as even more demanding. Khloe, a mother of a young child, for instance, shared that she had a difficult time after her baby was born because of the lack of personal space: “*I broke down at that point. The confinement, when it’s just the child.*” Such narratives underline the emotional labor of caregiving, and the perception that dogs—while demanding—allow for more personal autonomy than children. Dog care represents a form of manageable dependency, whereas childcare evokes a stronger sense of confinement and role engulfment.

Balancing work and care emerged as a recurring theme. Flexible schedules and home offices were often cited as essential for managing responsibilities. Iris, who did not have children, said, “*Home office is the best option. My dogs are right here with me. I can’t imagine a better job.*” Pamela, another childless participant, took her dog to work with her. “*I took her with me everywhere… There was some frustration because the bosses weren’t always happy that my dog was an extra responsibility. Later, … I was looking for a job where I could work from home or a dog-friendly office.*” These cases illustrate how care practices influence working life, showing that the emotional and logistical demands of pet care can shape women’s work trajectories in ways similar to those of motherhood.

In contrast, those with small children, such as Lucy, described tighter schedules and greater fatigue: “*This 24/7 readiness is incredibly taxing physically and mentally.*” Sarah, another mother, shared: “*I got swallowed up by all the stress, work, and perfectionism. I started going to therapy because I was showing anorexic symptoms… every night, I had panic attacks next to my child, and it was incredibly frightening.*” The comparison reveals that care intensity differs across dependents: dogs provide companionship and flexibility, while children introduce a more rigid and identity-defining caregiving role.

The interviews also reveal varying caregiving orientations, some of which appeared to extend across both dog and childcare. Importantly, we did not ask direct questions about parenting styles in the interview guide. All references to parenting approaches emerged spontaneously from participants’ narratives rather than as formal or self-identified parenting styles. Several mothers, such as Brooke and Rebecca, displayed an authoritative style, combining warmth with consistent expectations. For example, Brooke emphasized mutual respect and clear rules for both her dogs and children (“*Children must respect the dogs*”*)*, while Rebecca involved her children in the dogs’ daily care in a structured and supportive way. In contrast, some participants described more authoritarian tendencies, characterized by firm boundaries or a preference for obedience, although they did not explicitly report using the same corrective strategies across dogs and children. A few women expressed a more relaxed or indulgent stance, placing little emphasis on actively managing dog–child interactions. Khloe, for instance, noted that “*the dog loves the child, but could live without them,*” reflecting a more hands-off approach, even if this does not constitute a fully permissive caregiving style. The differences observed suggest that general caregiving philosophies often shape how women approach care across species.

Across groups, dogs were seen as structuring time but offering more flexibility than children. Walks and play provided exercise and emotional balance, while parenting required stricter, less negotiable routines. While dog ownership frequently expanded social networks, enabling spontaneous connections with other owners through walks, training schools, or online groups, parenthood, especially of young children, often reduced social opportunities and spontaneity, reinforcing family-centered routines.

### 3.3. Responsibility, Dependency, and Decision-Making

The practical demands aligned with how participants conceptualized responsibility across dogs and children. The distinction most consistently mentioned was the degree and duration of responsibility. Barbara, who did not have children, explained child parenting as a moral mission: “*It feels good to think I’m giving them the values I believe in.*”

Dog care, though serious, was perceived as less encompassing. For example, Lucy, a mother, said: “*You can leave a dog at home for three or four hours if you need to go somewhere. You just can’t do that with a small child.*” Ella, who was childless, emphasized that the responsibility for a child is higher than that for a dog: “*In terms of the level of commitment, a dog feels more comfortable… Having a child is on a different level. It’s longer-term, continuous.*” Fiona, another childless participant, demonstrated the difference quantitatively: “*I think if raising a child is ten on the responsibility scale, then a dog is about 0.01… Everything is your responsibility, and you can never blame anyone else, only yourself. That part is the same, and it’s incredibly annoying.*” In other words, while she emphasized the difference in the degree of responsibility, she concluded that the main common point in having a child or a dog is the attitude to raising a living being. Bianca, who did not have children, also reported a common point after acquiring her dog*:* “*During the first week or two, it was really hard, because the responsibility suddenly hit me. I had symptoms a bit like postpartum depression.*”

Participants also discussed financial and emotional responsibility. As Luna, a childless owner of elderly dogs, described the strains of aging and veterinary expenses: “*It’s really stressful, because if they’re sick, the vet can’t really tell what’s wrong… and since they’re old, it happens more and more often. You just end up throwing a lot of money out the window, and in the end, there’s nothing.*” Margaret, who was childless and considering motherhood, added: “*I walked through the children’s section at Tesco, then through dm [a shop selling baby products], and I said to myself that I’ll only have a child when I’m a multimillionaire. That’s one thing, the money, and the other main reason is the obligations, burdens, and difficulties of being a single parent.*” Solo caregiving further highlighted this difference. While many participants felt comfortable owning a dog on their own, several noted that having children requires a partner, stable housing, and financial security.

Adoption revealed another layer of responsibility. Over half of our participants adopted their dogs from shelters, describing satisfaction and moral reward. However, adopting a child was perceived as far more complex and risky. Emily, a mother, noted: “*It’s really better to have a dog because it’s less vulnerable, perhaps.*” On the other hand, Caroline, another mother, drew a parallel between dog and child adoption: “*I don’t understand why people keep having new children when there are already so many who don’t have families. It’s the same with dogs.*”

In summary, participants viewed both roles as requiring care and commitment, but child-rearing carried an enduring and morally weightier sense of obligation.

### 3.4. Social Relationships and Support

Dog ownership and parenthood shaped participants’ social worlds in contrasting ways. Some received negative feedback from pedestrians: Lucy, a mother: “It happened several times that people called me a stupid w****, asking why I don’t have a child instead… In Budapest, in N. street, walking three black dogs—well, that mostly attracted negative comments.” But usually, dogs served as social facilitators, creating opportunities for casual interaction. Lily, a mother, recalled: “*A little dog community formed… to this day we’re still friends on Facebook and Insta.*” Lisa, a childless participant, added: “*If we didn’t have a dog, I wouldn’t go anywhere except to the store or to buy supplies, and I wouldn’t meet anyone.*”

In contrast, parenting often leads to social contraction. Nora, a mother, compared the two: “*Because of the dog, we still meet up as often as before. Because of our little boy, much less*” Mothers frequently mentioned reduced spontaneity and a shift toward family-centered relationships. Single and childless women, however, found that dog ownership expanded their social networks, increased outdoor activity, and provided emotional continuity.

Several participants also discussed family attitudes. Khloe expressed that the dog provided emotional support for her father: “*The dog would retreat to ‘do therapy’ with my Dad*.” But dogs also provide support for their owners. After experiencing a miscarriage, Lily, now a mother, explained that her partner bought her a puppy, which she described as a form of grief therapy. Barbara, who did not have children, mentioned that “*Many times he takes care of me. Or at least I feel that he’s being nurturing, that he wants to take care of me.*” Luna also noted that her dog fulfils multiple roles for her, not only as a child, but also as a friend and, at times, as a maternal figure. Overall, the participants’ accounts highlight that caregiving is mutual between humans and dogs: while owners care for their dogs, the dogs also provide emotional support and comfort to their humans in times of need.

Some partners and relatives fully accepted the dog as a family member, while others found this attachment excessive. Margaret, a childless woman, shared: “*At first, I had conflicts with friends because I never went anywhere without my dogs. In the end, I just replaced the people who had a problem with this.*” Eva, who had a young child, described the same experience: “*Those who were bothered that my dog accompanied me everywhere eventually disappeared from my life.*” Sarah, a mother of one daughter, was left by her partner when she had multiple dogs. “*We used to live together, and since I’ve had these four dogs, he’s withdrawn.*” She also mentioned that since her daughter could not have a sibling, they had these dogs instead. Her parents initially had reservations: “*My parents think I’m a bit crazy because I have four dogs… But they help me and they adore them. Almost everyone around me has dogs, because if someone isn’t a dog person, they just don’t stay in my life.*” Overall, reactions to intensive pet caregiving were ambivalent. Some participants felt supported by their families and partners, whereas others recalled being judged for placing their dogs’ needs on par with those of children.

Parents often described a degree of bidirectional learning between child-rearing and dog care. Khloe noted that caring for her dog had taught her “*not to overdo the activities,*” a lesson she later applied to raising her child. Brooke described the reverse process: after becoming a mother, she related to her dogs “*in a healthier way,*” no longer treating them as child substitutes or reacting with excessive worry. Parents also emphasized the benefits dogs offered to their children. Veronica, for example, observed that growing up with a dog helped her son become calmer and more confident: “*he sees that no matter what he does or even if he gets angry, there is always someone who loves him unconditionally*”. Together, these accounts illustrate how experiences with dogs and children informed one another, shaping parents’ caregiving approaches across species.

However, some parents also expressed feeling sorry for their dogs. Caroline remarked, “*Most dogs get terribly hurt when a baby arrives in the family.*” Khloe added, “*I regret that I can’t spend enough time with him [the dog].*” These experiences show how caregiving, whether for dogs or children, reshapes social life, though in opposite directions: dogs generally foster social connectedness, while parenthood can limit it.

### 3.5. Life Course Perspectives on Caregiving

When reflecting on the future, participants emphasized how caregiving shaped their identity across the life span, life goals, aging expectations, and visions of family continuity. Several participants envisioned a future that included both dogs and (grand)children, as illustrated by this quote from Bella, a childless participant, who nonetheless imagined possible motherhood: “*A house by the water, dogs, a partner, and maybe children.*” Others focused on emotional and financial security rather than family expansion. Zoe, who did not have children, remarked: “*I hope that when I’m old, I’ll be in such good physical, mental, and spiritual shape that I’ll be completely able to take care of myself.*”

The lifespan difference between dogs and children influenced perceptions of time and legacy. Ella, who was childless, noted: “*It’s very hard for me that their life isn’t measured in human years… it constantly weighs on me that they’re going to pass away.*” The awareness of dogs’ shorter lifespans generated complex emotions such as anticipatory grief and reflections on impermanence. Brooke shared personal stories: “*I have a friend who committed suicide after her dog died, and another who has been mourning her dog for four years and simply cannot get over the loss.*” Conversely, parents often described children as long-term investments in both care and identity, often accompanied by concern about their own aging.

Some participants saw dogs as companions for midlife or later years, offering emotional stability without the long-term dependency of raising a child. For childless respondents, dogs represented continuity and companionship, filling a caregiving role without altering their life trajectory. For mothers, dogs complemented family life, easing transitions as children grew more independent. They also tended to frame their children as sources of long-term legacy and purpose but acknowledged the emotional strain of continuous caregiving. Overall, caregiving, whether for dogs or children, emerged as a defining element of identity and emotional continuity across the life course, thus shaping participants’ future outlook and sense of aging.

Table 3 summarizes the main insights obtained from the thematic analysis.

## 4. Discussion

This qualitative study explored how women with and without children construct the meanings of caregiving for dogs and children. The findings show that dog ownership can evoke parenting-like emotions and behaviors, while species- and role-based distinctions remain important. These narratives expand our understanding of caregiving and emotional bonds beyond the human family and illustrate how pet care provides a context in which nurturing, responsibility, and emotional reciprocity are enacted in everyday life.

Participants often anthropomorphized their dogs, calling themselves “mom” or “dad,” or referring to their dogs as “my baby” or “my son.” These terms demonstrate the extension of parental metaphors to non-human dependents, reflecting what previous studies have termed pet parenting [13,15,16,17,19,20,62,64]. Through this framing, dogs were positioned as emotionally significant family members rather than possessions or leisure companions. These expressions are in line with psychological and neurobiological evidence showing that the human–dog bond can activate caregiving systems similar to those involved in parenting [52].

At the same time, participants’ narratives revealed an important difference from childcare: dog care was rarely described as one-directional. Dogs were seen as not only receiving care but also giving emotional support and comfort to their owners, offering a sense of calm, companionship, and unconditional acceptance. This perceived reciprocity highlights that, in contrast to the parent–child relationship, the emotional bond between humans and dogs involves both giving and receiving care. This may help explain why such relationships are often described as uniquely stabilizing and restorative.

Our results also underline how parental status shapes these experiences. For many childless women, dogs were central figures in their emotional lives. They often described the dog as a “first child,” a “zero child,” or as someone who filled a space where a child might have been. In these cases, dog caregiving could serve as a symbolic rehearsal for possible motherhood or as a way of coping with involuntary childlessness. In contrast, mothers tended to integrate dogs into an already existing family system and more often spoke of balancing the needs of children and dogs. They expressed strong affection for their dogs but clearly prioritized their children in situations of conflict. This pattern echoes the moral hierarchy of caregiving described in earlier research [16,17,34,41].

Consistent with previous studies [12,35,64,65], participants described dogs as sources of unconditional love, companionship, and comfort. Such relationships fulfill core psychological needs for attachment and caregiving that were once primarily met through human community ties [6,66,67,68]. For some women without children, dog ownership offered a flexible and emotionally rewarding way to express nurturing behavior in life circumstances that delayed or complicated parenthood. This resonates with recent discussions on how pet caregiving can provide both emotional fulfillment and a sense of identity continuity in changing social contexts [6,15,28,62]. For mothers, by contrast, dogs often supplemented rather than replaced human caregiving roles. Dogs were described as additional dependents, companions for children, or emotional supports within family life rather than substitutes for children.

Participants’ accounts of structured routines, such as feeding, walking, and shared play, show that caregiving practices for dogs can resemble those used in parenting young children [35,65,69]. These routines reflect the translation of parental time structures into the domain of pet care and suggest that caregiving behaviors are organized around similar logics of responsibility, predictability, and affection. However, there was a clear difference between groups. Childless women frequently framed dog-related routines as central organizing structures in their daily lives. For mothers, child-related schedules were more dominant, and dog routines were often fitted around children’s needs. Across the sample, dog care was consistently described as more flexible and compatible with modern urban life. While pets frequently function as social catalysts, facilitating interaction and community building [6], parenting was more often associated with constraints and reduced spontaneity. The possibility of adjusting routines or temporarily delegating responsibility constructed pet care as a more voluntary, self-regulated commitment rather than a moral obligation. This distinction between dog care and childcare illustrates how women manage competing expectations of autonomy and care, balancing self-realization with emotional connectedness [15,19]. Overall, these findings indicate that dog caregiving occupies an intermediate position between self-care (activities that support one’s own physical and emotional well-being) and parenting, as it demands structure and empathy, while still allowing autonomy and recovery.

The coexistence of warmth and structure in these caregiving narratives echoes the concept of authoritative parenting, originally proposed by Baumrind [70] and later adapted for canine contexts [39,40,43,71]. Most respondents described an emotionally responsive yet rule-guided relationship with their dogs, combining affection with consistent expectations. This pattern mirrors what Volsche and Gray [42] identified among “dog moms”: a caregiving orientation that blends empathy with structure, allowing emotional closeness without losing behavioral boundaries. Our data suggest that women often carry general caregiving philosophies across species, even though we did not ask direct questions about parenting styles. For childless women, these orientations primarily shaped dog–human interactions. For mothers, they applied to both children and dogs but with different weights and priorities.

Despite these parallels, participants clearly distinguished dog caregiving from human parenting in moral and temporal terms. Parenting was described as a lifelong, socially sanctioned duty involving moral education and the transmission of values. Dog ownership, in contrast, was perceived as a meaningful yet temporally bounded relationship. Financial and emotional responsibilities, such as veterinary bills, training costs, and distress during illness, were acknowledged as parallel to parenting concerns, but the perceived stakes were lower. The finite lifespan of dogs and the absence of long-term social obligations made pet care feel intense yet manageable. This distinction supports previous research indicating that, although dogs may evoke caregiving instincts similar to those of parenting, the perceived moral weight and permanence of human child-rearing remain unmatched [16,34,68]. The contrast between children and dogs underlines how caregiving toward animals offers emotional engagement without the existential weight of child-rearing [72]. However, it should be noted that our respondents had relatively young children, with the oldest child aged 13. Owens and Grauerholz [20] found that parents of young children report feeling substantially greater commitment and responsibility toward their children than toward their dogs, whereas parents of older children are more likely to emphasize the similarities.

Social reactions to intensive pet caregiving reflected broader cultural tensions. Some participants reported understanding partners and family members, while others faced criticism for “treating dogs like children.” These responses mirror public debates about intensive motherhood [73] and the moral evaluation of women’s caregiving choices. Pet parenting, like mothering, is subject to social scrutiny, especially when it challenges normative hierarchies of care. Childless women, in particular, reported being judged for investing heavily in their dogs instead of having children, whereas mothers more often faced criticism for “spoiling” animals or dividing their attention between dogs and children. By negotiating legitimacy through expressions of responsibility and affection, participants positioned themselves as ethical caregivers within evolving frameworks of femininity and family. The common emergence of ideals of “responsible dog ownership” and “good motherhood” reveals how both domains are shaped by gendered expectations of devotion, self-sacrifice, and emotional availability [20,42].

The future-oriented aspects of caregiving further demonstrated its role in constructing identity and continuity. Many women described dogs as emotional anchors and companions in middle or older age, providing stability and helping them manage loneliness and stress [74,75,76]. This finding aligns with studies showing that pets mitigate isolation and support well-being across the lifespan [77]. Differences by parental status emerged again: childless women often described dogs as central companions in later life and as sources of continuity in the absence of children. Mothers, by contrast, tended to see their children as bearers of legacy and long-term purpose but also acknowledged the exhaustion of continuous caregiving and the relief they anticipated as children grew more independent. The awareness of dogs’ shorter lifespans evoked ambivalent feelings: anticipatory grief but also gratitude for the intensity of shared time. For childless women, this temporariness seemed to make caregiving more emotionally sustainable, offering intimacy without the permanent demands of parenthood. Similar to earlier findings [19,20]. These accounts suggest that pet care can satisfy core human needs for nurture and belonging while fitting flexibly into contemporary life structures.

Regarding the biological mechanisms, participants often referenced sensory and interactive cues (e.g., ‘puppy-eyes’ gaze, tactile and olfactory affection, shared activity) that align with ethological mechanisms such as the baby-schema effect [78,79,80] and expressive facial musculature linked to human responsiveness [81]. This may further help explain why dogs, more than other companion animals, often become embedded in family systems and evoke parent-like emotions.

Several participants described their dogs as “child-like,” “vulnerable,” or “needing protection,” and these perceptions align with well-documented ethological mechanisms that elicit human caregiving responses. Infant-like morphological features in dogs—such as large eyes, rounded faces, and small noses—activate the “baby schema” effect, which evokes nurturing emotions and prosocial behavior in humans [82,83,84]. Research also shows that dogs’ expressive facial movements, particularly the inner-brow raise associated with the “puppy-eyes” expression, increase human responsiveness and adoption likelihood [81]. In addition, dependent behaviors such as help-seeking gaze in problem-solving situations can reinforce perceptions of dogs as needing guidance or protection [60]. These mechanisms may help explain why several women in our sample intuitively described their dogs as resembling young children, and why caregiving impulses can become so emotionally salient, especially in the case of brachycephalic breeds that elicit particularly strong nurturing responses [85,86].

Taken together, these findings indicate that caregiving toward dogs and children shares emotional foundations rooted in attachment, responsibility, and social meaning. Yet the differences, particularly in moral obligation, social recognition, and permanence, mark the limits of interspecies comparison. Dog caregiving occupies an intermediate position between self-care and parenting: it requires structure and emotional investment but preserves personal autonomy. As such, it may represent an adaptive expression of care in societies where traditional family forms and gender roles are changing [6,28]. Recognizing the continuities between human and animal caregiving can enrich theoretical models of attachment and identity while informing welfare and housing policies that acknowledge the social significance of companion animals. Rather than viewing pet care as an alternative to human reproduction, these results highlight its role within the broader continuum of caregiving that defines human social life.

While this study offers in-depth insight into the lived experiences of women who care for both children and dogs, it is limited by its small, relatively homogeneous sample of urban, highly educated Hungarian participants. The findings, therefore, reflect a specific sociocultural context in which pet parenting and motherhood intersect within European, middle-class lifestyles. Future research should adopt more diverse and comparative approaches. Analyzing the narratives of participants from different cultural, geographic, and socioeconomic backgrounds would allow us to examine how structural factors, such as housing conditions, gender norms, or welfare policies, shape caregiving meanings and practices. Longitudinal and mixed-method studies could further explore how caregiving priorities evolve over time, particularly as family configurations, professional roles, and dogs’ life stages change. Comparative studies involving men or non-binary caregivers would also provide valuable perspectives on how gendered expectations influence interspecies caregiving. Finally, integrating behavioral or physiological measures could complement interview data and deepen understanding of the emotional and health-related impacts of dog–human caregiving relationships.

## 5. Conclusions

By comparing women’s experiences of caring for dogs and children, this study reveals deep emotional and behavioral similarities across species. Dogs can function as companions and recipients of caregiving behaviors that closely resemble parenting, extending empathy and responsibility beyond the human family. Importantly, participants also described caregiving as reciprocal: dogs not only receive care but also provide emotional support, comfort, and a sense of security to their owners. This mutual exchange of care further explains their central role in women’s emotional lives. Yet, the contrast between children and dogs underlines how caregiving toward animals offers emotional engagement without the existential weight and moral permanence of child-rearing. Rather than replacing children, dog caregiving represents a flexible form of nurturing that aligns with contemporary social realities. These findings contribute to a growing understanding of the human–animal bond as a vital and evolving expression of caregiving in modern society.

## Figures and Tables

**Table 1:** Demographic data of the respondents.

Pseudonym	Age	Education	Partnership Status	Children (Ages)	Dogs (Number)
Alice	34	MA/MSc	Married	3	2
Barbara	27	BA/BSc	In a relationship	-	1
Bella	33	BA/BSc	Single	-	1
Bianca	30	BA/BSc	Married	-	1
Brooke	43	BA/BSc	Married	9, 10	2
Caroline	39	MA/MSc	Married	5	1
Dolores	30	MA/MSc	In a relationship	-	4
Ella	33	BA/BSc	In a relationship	-	1
Emily	47	BA/BSc	Married	13	1
Eva	38	MA/MSc	Married	2	1
Fiona	38	BA/BSc	Married	-	2
Hailey	33	BA/BSc	Married	1	1
Iris	33	MA/MSc	Single	-	1
Khloe	40	MA/MSc	Married	2	1
Lily	39	High school diploma	Married	2	1
Lisa	32	High school diploma	In a relationship	-	1
Lucy	42	MA/MSc	Married	2	3
Luna	29	MA/MSc	In a relationship	-	1
Margaret	27	MA/MSc	In a relationship	-	2
Maya	30	BA/BSc	Married	-	1
Nora	32	MA/MSc	Married	2	1
Pamela	26	BA/BSc	In a relationship	-	1
Rebecca	29	MA/MSc	Married	4	2
Sarah	47	MA/MSc	In a relationship	12	4
Sophie	27	BA/BSc	Single	-	1
Veronica	40	BA/BSc	Married	9, 11	1
Willow	46	MA/MSc	Single	-	1
Zoe	36	Post secondary diploma	Married	-	1

**Table 2:** Theme categories.

Theme	Key Codes	Comparative Focus
Emotional meanings and motivations of caregiving	Joys, challenges, parental language, “Dog instead of children?”	Emotional fulfillment and caregiving identity toward dogs vs. children
Practical caregiving and daily routines	Daily routine, Work balance, Dog–child routines	How dogs and children structure daily activities and time use
Responsibility, dependency, and decision-making	Adoption, Solo parenthood, Responsibility	Perceived weight of care and autonomy differences between dogs and children
Social relationships and support	Family and friends attitudes, Social life, Workplace context	How caregiving affects social ties and social support systems
Life course perspectives: Future, identity, and aging	Climate concerns, Future outlook, and Later life	How future plans, identity, and aging are tied to pet and child care

**Table 3:** Summary and main insights of themes.

Theme	Main Insight
Emotional meanings	Dogs and children evoke strong nurturing emotions; maternal language is common, but boundaries are recognized.
Daily routines	Both require structure and consistency; dogs provide flexibility and balance, children more rigidity.
Responsibility	Parenting is seen as lifelong and moral; dog care is serious but limited and more autonomous.
Social support	Dogs facilitate social contact, and caregiving can be mutual; children shift social focus toward family and reduce spontaneity.
Life course perspective	Dogs offer companionship and emotional continuity; children represent long-term legacy and responsibility.

## Data Availability

The qualitative interview data supporting the findings of this study are available from the corresponding author upon reasonable request.
